# Balancing selection on the complement system of a wild rodent

**DOI:** 10.1186/s12862-023-02122-0

**Published:** 2023-05-25

**Authors:** Mridula Nandakumar, Max Lundberg, Fredric Carlsson, Lars Råberg

**Affiliations:** Department of Biology, Lund University, Lund, Sweden

**Keywords:** Complement system, Pathogen-mediated selection, Balancing selection, Bank voles, Myodes glareolus

## Abstract

**Background:**

Selection pressure exerted by pathogens can influence patterns of genetic diversity in the host. In the immune system especially, numerous genes encode proteins involved in antagonistic interactions with pathogens, paving the way for coevolution that results in increased genetic diversity as a consequence of balancing selection. The complement system is a key component of innate immunity. Many complement proteins interact directly with pathogens, either by recognising pathogen molecules for complement activation, or by serving as targets of pathogen immune evasion mechanisms. Complement genes can therefore be expected to be important targets of pathogen-mediated balancing selection, but analyses of such selection on this part of the immune system have been limited.

**Results:**

Using a population sample of whole-genome resequencing data from wild bank voles (n = 31), we estimated the extent of genetic diversity and tested for signatures of balancing selection in multiple complement genes (n = 44). Complement genes showed higher values of standardised β (a statistic expected to be high under balancing selection) than the genome-wide average of protein coding genes. One complement gene, *FCNA*, a pattern recognition molecule that interacts directly with pathogens, was found to have a signature of balancing selection, as indicated by the Hudson-Kreitman-Aguadé test (HKA) test. Scans for localised signatures of balancing selection in this gene indicated that the target of balancing selection was found in exonic regions involved in ligand binding.

**Conclusion:**

The present study adds to the growing evidence that balancing selection may be an important evolutionary force on components of the innate immune system. The identified target in the complement system typifies the expectation that balancing selection acts on genes encoding proteins involved in direct interactions with pathogens.

**Supplementary Information:**

The online version contains supplementary material available at 10.1186/s12862-023-02122-0.

## Background

**Fig. 1:**
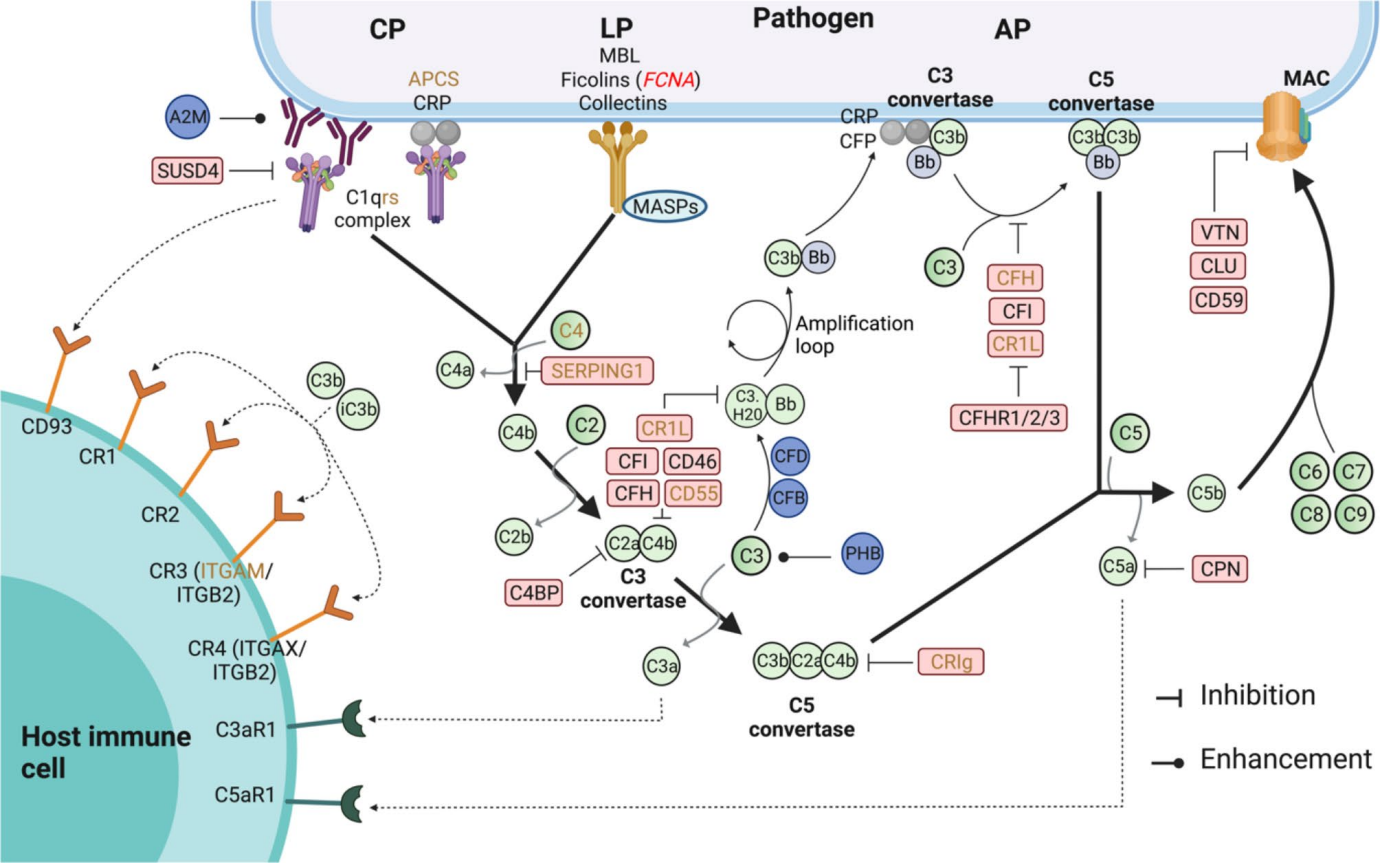
Overview of the complement cascade. The cue to signal the complement cascade can be varied and helps determine the pathway that is activated. The classical pathway (CP) is primarily triggered when C1Q recognises and binds to the Fc region of antibodies or pentraxins bound to antigens. The lectin pathway (LP) is initiated when lectins of the complement system bind carbohydrates on cell surfaces. The alternative pathway (AP) is active constantly at low levels due to spontaneous hydrolysis of complement component C3, leading to indiscriminate C3b deposition on nearby cell surfaces. This is immediately cleared by regulators of complement activation (RCA) present on host cells, preventing complement activation. Microbes lack RCA proteins and thereby activate the alternative pathway. Thus, only the lectin pathway requires specific recognition of MAMPs. In all cases, C3 convertases are produced once the pathway is triggered which in turn cleaves complement component C5, with all pathways converging at this stage. Ultimately, a membrane-attack complex (MAC) is assembled and inserted on the pathogen surface, and this pore lyses the pathogen cell. In addition, complement activation leads to opsonization and activation of other inflammatory and immune pathways. The figure is for representative purposes and only a single role for complement proteins with multiple functions is presented here. Text in brown indicate genes that were not included in this study. Text in red indicate the gene with signature of balancing selection in the bank vole. Created with BioRender.com.

The complement system is an evolutionarily ancient branch of innate immunity that occurs in both invertebrates and vertebrates. In mammals, it consists of more than 40 soluble and membrane-bound proteins. It provides manifold utility in immune defence, including recognition, opsonization and killing of pathogens, clearance of apoptotic cells, induction of inflammation, and priming of other innate and adaptive responses [1, 2]. Depending on the initial cue, the complement cascade can be activated via the classical, lectin and alternative pathways. All three pathways converge at complement component 3 (C3) and may ultimately lead to the insertion of a membrane-attack complex (MAC) into the surface of the pathogen to cause its lysis, along with other effects in the host (see Fig. 1 for details).

Immune genes, in particular those encoding proteins that are involved in direct and specific interactions with pathogen ligands, are considered prime candidates for host-pathogen coevolution [3–5]. Complement proteins interact with pathogen molecules in multiple ways. First, some complement proteins act as sensors of pathogens by recognising microbe-associated molecular patterns (MAMPs), triggering activation of the complement cascade. Such sensors include the pattern recognition molecules (PRMs) of the lectin pathway (mannose-binding lectins, collectins, and ficolins) and C-reactive protein (CRP). For instance, the PRMs mannose-binding lectins (MBL) recognise carbohydrates on pathogen cell surfaces, activating the lectin pathway [6]. The complement system can also be activated in other ways, including by recognition of antibodies deposited on pathogens (classical pathway) and by “missing self-recognition” (alternative pathway), but these do not involve direct and specific interactions between complement proteins and pathogen ligands (see Fig. 1 for details).

Second, proteins of the complement system are often targets of pathogen immune evasion factors. Common strategies employed by pathogens include direct inhibition of complement proteins and the recruitment or mimicking of endogenous regulators of complement activation (RCA) [7]. For example, species of the Lyme disease spirochaete *Borrelia burgdorferi sensu lato* complex employ a multi-pronged (and often redundant) approach to prevent destruction by the complement system [8, 9]. Multiple *Borrelia* proteins belonging to the complement regulator-acquiring surface proteins (CRASPs) recruit RCA protein complement factor H (FH) and prevent complement activation [10]. *Borrelia* also produce outer surface proteins and complement protein mimics that directly interact with and inhibit complement proteins or MAC formation [11–13]. Other pathogens such as *Streptococcus pyogenes, Neisseria meningitidis* and *Staphylococcus aureus* use similar strategies to hijack the complement system to their advantage and escape killing [14–17].

As described above, many of the complement proteins are involved in direct physical and antagonistic interactions with specific pathogen ligands. As such, complement genes can be expected to be involved in coevolution with pathogens [4, 18, 19]. This view is supported by studies showing complement system genes to be under positive selection across species (i.e., selection driving divergence between species), as demonstrated by genome-wide scans as well as analyses of specific genes. For example, a study looking at genome-wide patterns of positive selection across different mammalian species identified genes involved in the complement system to be significantly enriched for targets of positive selection [20]. Similarly, a comprehensive study looking at positive selection on the complement system across primates identified positively selected sites in numerous complement genes, primarily located in protein domains at the interface of pathogen interactions [5].

Besides positive selection across species, host-pathogen interactions like antagonistic coevolution can also lead to balancing selection, resulting in high genetic diversity within species [21]. However, only a handful of studies have investigated balancing selection in specific complement genes. In the case of human gene *MBL2*, which encodes a PRM, high diversity in its sequence is observed; however, whether this diversity is a consequence of balancing selection is still debatable [22–24]. Another study looking at human complement gene *C6*, which is often a target of immune evasion by pathogens, found evidence of balancing selection across different human populations [25]. However, a comprehensive analysis examining balancing selection across all complement genes has not been attempted in any species.

In this study, we investigate the extent of balancing selection in complement system genes of bank voles (*Myodes glareolou*s) using whole-genome resequencing data. We first identify complement genes with signatures of balancing selection in coding and/or noncoding regions as indicated by BetaScan2 [26]. Second, for genes picked up by BetaScan2 we used a classical neutrality test (the HKA test;[27]) to test for signatures of balancing selection across the whole protein-coding sequence (CDS) [28]. Finally, we look for factors influencing which complement genes have signatures of balancing selection as indicated by BetaScan2. In this case, we hypothesise that complement genes encoding proteins that participate in direct and specific interactions with pathogens, such as those involved in pathogen recognition (i.e., PRMs) or targets of immune evasion, are more likely to be under balancing selection (henceforth collectively referred to as “candidates of balancing selection”) compared to those that are not involved in similar interactions.

## Results

We curated complement genes from literature and public databases, of which 44 complement genes were annotated in our bank vole genome assembly that could be used for analyses of nucleotide diversity and balancing selection. A few additional complement genes were found in the assembly but were excluded due to annotation issues, insufficient coverage as compared to the mouse CDS (< 50%) or poor alignment with mouse CDS (Additional File 1: Table S1).

We used whole genome resequencing data from 31 bank voles to provide estimates of genetic diversity and balancing selection. To shortlist genes with signatures of balancing selection, we adopted an outlier analysis for the β estimates provided by BetaScan2 [26]. β is a summary statistic based on allele frequency correlations between neighbouring SNPs, where elevated values are indicative of balancing selection due to genetic hitchhiking and drift of neutral variants. We standardised the β values with locus-specific mutation rate (β_std_), which was calculated in 2 kb windows across the whole genome (see Methods for details). The maximum value of β_std_ in each gene was identified (β_std.max_). Four genes (*CFHR1, FCNA, ITGAX* and *HC;* Table 1) were outliers for β_std.max_, with β_std.max_ ≥ 95th percentile of a set of control genes (n = 8465; 95th percentile of control β_std.max_ = 7.03).

**Table 1:** Diversity and balancing selection metrics for the complement genes with β_std.max_ ≥ 95th percentile of control genes (7.03). Gene in bold indicates consistent signature of balancing selection

Gene	CDS length	No. of segregating sites (S)	Nucleotide diversity (π)	Selection parameter (k in MLHKA test)	p-value (MLHKA test)	Highest β in gene (β_std.max_)	Percentile rank of β_std.max_
***FCNA***	1005	28	0.0103	2.72	0.024	7.89	97.2
*ITGAX*	3468	34	0.0038	1.04	1	13.46	99.9
*CFHR1*	822	7	0.0015	1.23	1	8.40	97.9
*HC*	4545	32	0.0025	1.12	1	7.15	95.4

We then used the maximum likelihood Hudson-Kreitman-Aguadé test (MLHKA) [27], which tests for selection by comparing polymorphism and divergence to a neutral scenario. We used MLHKA to test for signatures of balancing selection across full coding sequences. We limited the MLHKA tests to the four genes that were outliers of β_std.max_. Of these, only *FCNA* (ficolin A) was significant by MLHKA, with selection increasing the diversity by 2.72x (as indicated by the selection parameter k; Table 1). We thus focused further analyses on this gene.

**Fig. 2:**
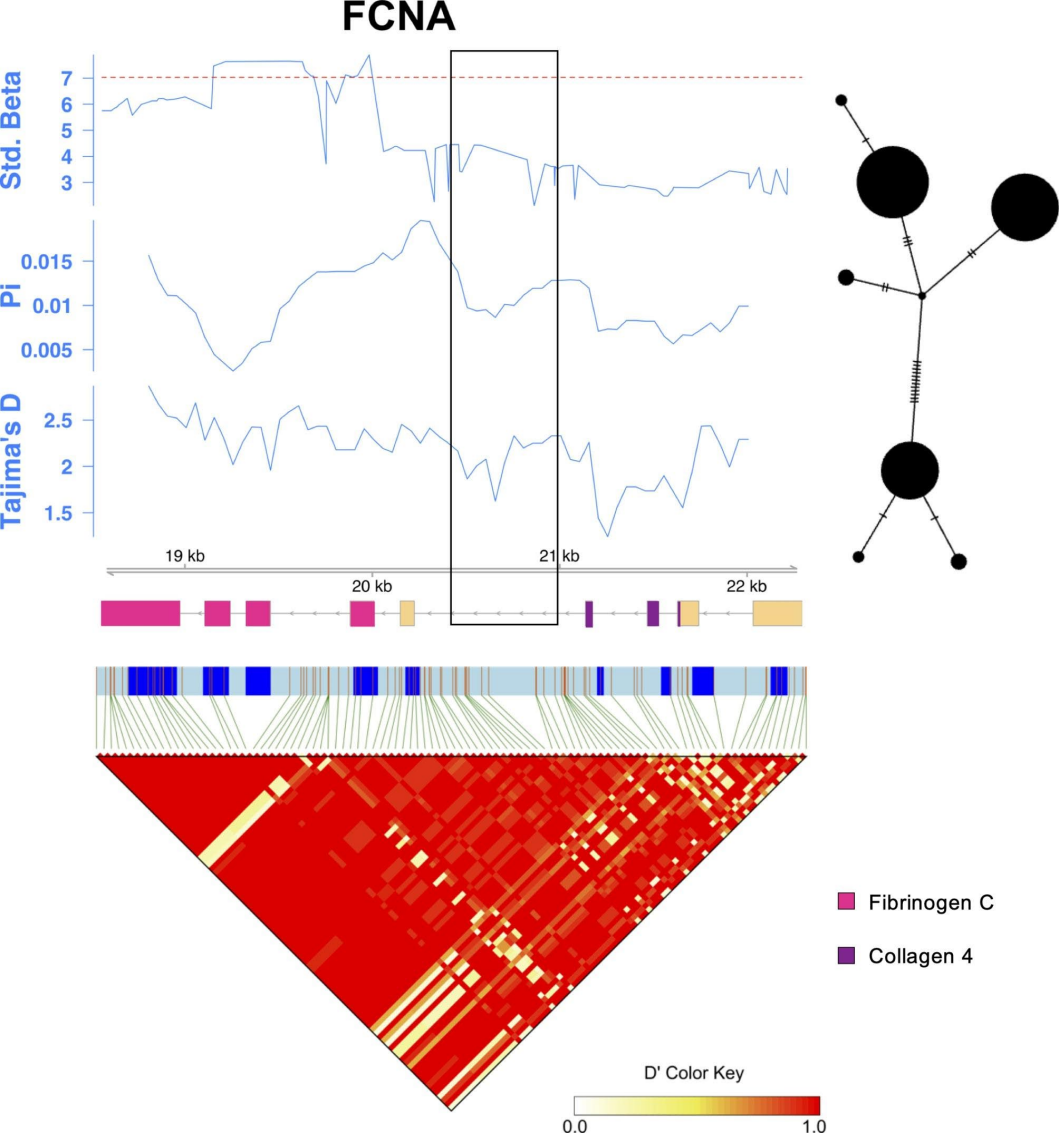
Sliding window analysis of β_std_, π, and Tajima’s D, haplotype network, and LD plot for *FCNA.* Red dashed line indicates the 95th percentile of β _std max_ of control genes. The haplotype network was constructed from the region marked with black square. Gene structure below sliding window plots show protein domains as predicted by Pfam [33]. The LD plot was constructed for the whole gene using D’, with LD blocks shown. D’ refers to the normalised values of the coefficient of LD, with high values indicating strong LD. Gene structure corresponding to the LD plots show introns in light blue and exons in dark blue

To identify gene regions that were targets of balancing selection in *FCNA*, we plotted β_std_, nucleotide diversity (π), and Tajima’s D along the gene, and calculated linkage disequilibrium (LD) using the normalised LD coefficient D’ (Fig. 2). The parameters β_std_, π and Tajima’s D primarily reflect long-term balancing selection and are expected to be elevated under this scenario [29–31]. Tajima’s D is based on comparing two different estimates of genetic variation; nucleotide diversity and number of segregating sites. Tajima’s D is sensitive to both selection and demographic processes: D > 0 when there is an excess of intermediate frequency alleles as a result of balancing selection or demographic processes like population contraction; D < 0 when there is an excess of rare alleles, for example, after a selective sweep or population expansion; D = 0 under neutral evolution and constant population size [32]. To visualise the number and frequency of different haplotypes present in the population, we also constructed a haplotype network bordering the most polymorphic region using the whole-genome resequencing data complemented with Sanger sequencing data from an additional 30 bank voles. Under balancing selection, one would expect to see at least two well-separated haplotype groups.

*FCNA* showed high values of π and Tajima’s D almost throughout the entirety of its short length (Fig. 2). Twelve SNPs in *FCNA* had β_std_ above the 95th percentile of β_std.max_ of a set of 8465 control genes, all in the region from exons 6 to 8. It is notable that these exons encode the fibrinogen C domain, which is known to recognise carbohydrates with N-acetylation markers. As a possible consequence of its short length, the whole gene formed a single LD block. A haplotype network based on ~ 700 bp covering a part of the peak in nucleotide diversity showed two well separated haplotype groups. Similar analyses of the other three genes that were outliers for β_std.max_ are presented in Supplementary Figure S1 (Additional File 2).

**Fig. 3:**
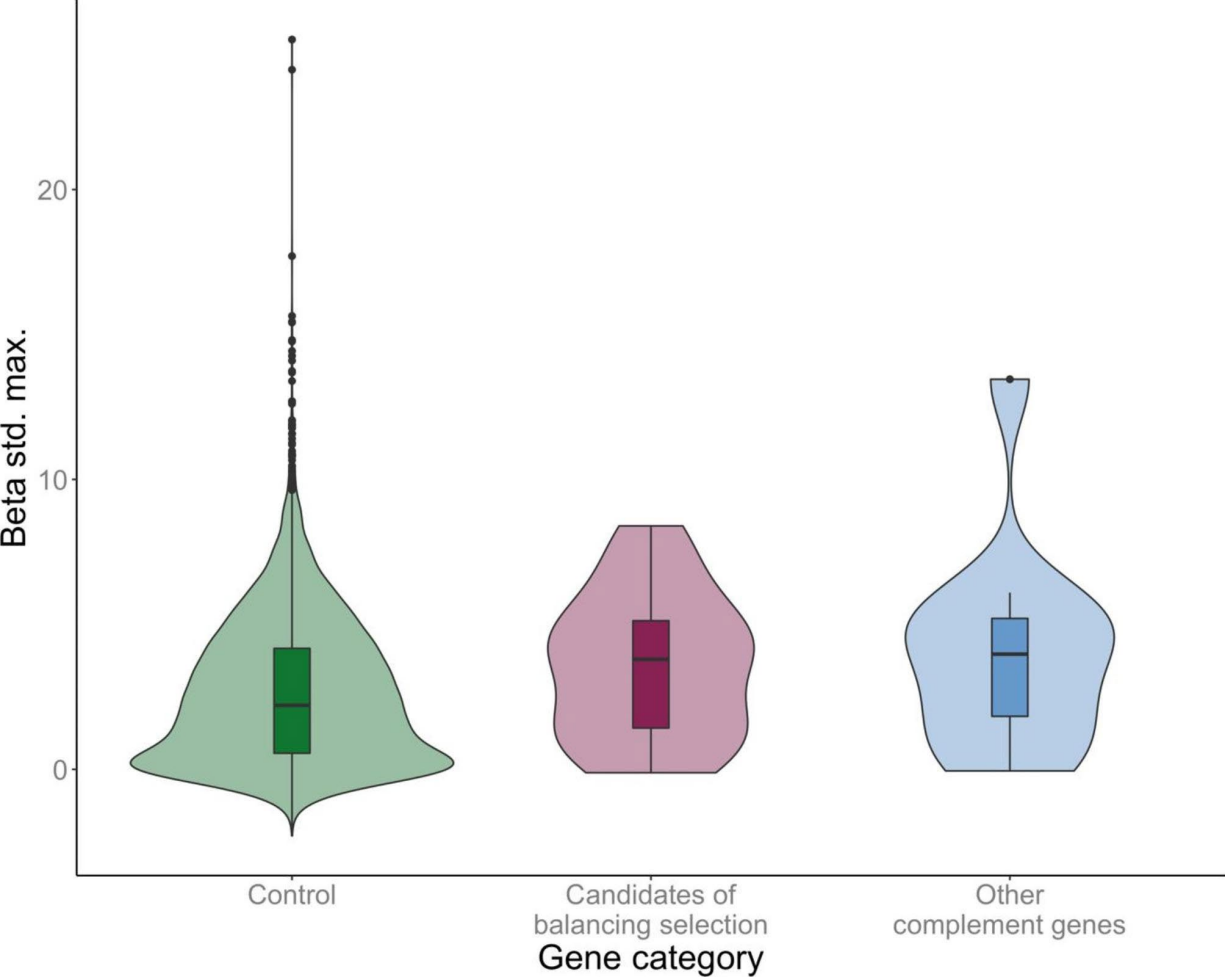
Violin plots of β_std.max_ for non-complement control genes (n = 8465), complement genes that are candidates of balancing selection (i.e., involved in pathogen recognition or are targets of immune evasion; n = 25), and other complement genes (n = 12)

We hypothesised that complement genes that are engaged in direct and specific interactions with pathogens, such as those activating the complement cascade by recognising pathogens or are subject to immune evasion, would be more likely to evolve under balancing selection. To test this, we compared β_std.max_ of complement genes that were candidates of balancing selection (i.e., complement genes encoding PRM/targets of immune evasion; n = 25) and other complement genes (n = 12). There was no difference in β_std.max_ between these two categories of complement genes (Mann-Whitney U test: p = 0.86; Fig. 3). However, complement genes overall (n = 37) showed elevated β_std.max_ as compared to control genes (Mann-Whitney U test test: p = 0.014).

## Discussion

In this study, we investigated the extent of balancing selection in complement system genes of bank voles. Based on analyses of whole gene sequences, we found signatures of balancing selection in four complement genes, as indicated by the β_std.max_ outlier analysis (*FCNA, CFHR1, HC, ITGAX*). However, only *FCNA* showed a significant departure from neutrality as assessed by the MLHKA test. *FCNA* also showed divergent haplotype groups at intermediary frequencies. Taken together, this is consistent with evolution under balancing selection at *FCNA*. We acknowledge the risk for false positives when testing for signatures of selection in multiple genes [34] and note that the MLHKA test for *FCNA* does not pass the Bonferroni-adjusted threshold (which would be α = 0.05/37 = 0.0014). However, the fact that both BetaScan2 and the MLHKA test, which are based on partly different information, detected signatures of balancing selection in *FCNA* indicates that it is not a false positive. Ultimately, tests for signatures of selection should be complemented with functional analyses of alternative alleles [35].

*FCNA* (ficolin A) in bank voles is orthologous to *FCN2* in humans, with both genes encoding a lectin PRM that recognises carbohydrates. Information from human and mice indicate that it forms homotrimers that preferentially recognise N-acetylated carbohydrates such as N-acetylglucosamine (GlcNAc) and N-acetyl galactosamine [36–38]. In microbes, GlcNAc is fundamental to forming chitin in fungal cell walls and the peptidoglycan layer of bacterial cell walls, a component abundant in gram-positive bacteria and present to a smaller extent also in gram-negative bacteria. In conjunction with this information, *FCNA* knockout mice are impaired in their ability to respond to certain pathogens such as the gram-positive bacteria *Streptococcus pneumoniae* and the fungus *Aspergillus fumigatus* [39–41], highlighting the importance of the protein to the immune response.

Previous studies of balancing selection on complement system genes are few and have been limited to humans. *FCNA*, is functionally analogous to *MBL2*, which is contested to be under balancing selection in humans [22–24]. However, unlike in humans where there is some evidence for balancing selection on *C6* [25], we were unable to corroborate the presence of balancing selection in any of the bank vole genes encoding complement components deposited on pathogens.

We also looked for general differences between different categories of complement genes. Balancing selection due to pathogen-mediated pressure is expected to act on genes that are the focal points of host-pathogen interactions. A previous study of balancing selection in bank voles by Lundberg et al. [19] analysed genes of innate immune signalling pathways. Genes encoding Pattern Recognition Receptors (PRR) – specifically, those recognising microbial cell wall components – displayed higher β_max_ values than genes involved in downstream signal transduction. Similarly, a study by Cagliani et al. [5] on the complement system of primates found that many genes under positive selection directly interacted with pathogens. Consequently, we hypothesised that certain complement genes (those involved in pathogen recognition or are targets of immune evasion), would show increased signatures of balancing selection. Complement genes overall showed higher β_std.max_ than control genes. However, in contrast to patterns observed in analyses of PRR signalling pathways [19], complement genes involved in direct interactions with pathogens did not have higher β_std max_ than other complement genes.

Analysis of LD can be informative in understanding the time since a selection event. When an advantageous allele arises *de novo* in a population or is favoured from standing variation, it increases in frequency and can often sweep variants at neighbouring linked neutral sites, reducing heterozygosity in these regions. During the early stages of balancing selection, this results in strong LD over large genomic regions, due to insufficient time for recombination to act [31]. However, given sufficient evolutionary time, the frequency of the advantageous allele stabilises at an intermediate level and the regions of LD begin to reduce due to recombination. In our data, we notice that *FCNA* shows LD, despite signatures of balancing selection indicated by MLHKA and BetaScan2, which are both designed to detect intermediate to ancient selection events [29, 30]. The extremely short length of *FCNA* (around 3 kb) can potentially explain the lack of recombination observed for this gene.

The targets of balancing selection in immune genes are often expected to occur in coding regions, specifically at sites that are important for interaction with pathogens [3, 5]. In the case of *FCNA*, the peak in β_std_ spans three exons coding for the ligand binding region. This exemplifies the expectation that targets of balancing selection within the immune system occur in coding regions that recognise pathogens. However, further analyses are required to attribute the function of polymorphisms found in this gene.

## Conclusions

In this study, we demonstrate signatures of balancing selection on *FCNA*, a complement gene encoding a protein involved in direct interactions with pathogens. Further studies to determine the functional effects of the different *FCNA* alleles are warranted. The present study is the most comprehensive analysis of balancing selection in the complement system carried out thus far, and adds to the growing evidence that balancing selection is an important evolutionary force not only on genes of the adaptive immune system (e.g. MHC and other genes involved in antigen presentation; [42, 43]), but also on different components of the innate system, such as pattern-recognition receptors, defensins, antiviral restriction factors, and the complement system [19, 44–47].

## Methods

### Complement system genes

The primary source for complement system genes was the KEGG [48] pathway “Complement and coagulation cascade” (mmu:04610). Genes pertinent to only the complement system were retained, based on the review by Ricklin et al., [1]. To expand the list to more recently annotated genes, additional information from the Mouse Genome Database (MGD; [49]) in Mouse Genome Informatics (MGI) was included, after verification of function from literature. In total, n = 60 genes were selected. Orthologs of these mouse genes that were annotated in our bank vole genome assembly [19] and covered at least ~ 50% of the mouse coding sequence were retained for analysis. For four genes, annotations were curated manually in Web Apollo [50]. This yielded 44 bank vole complement genes for further analyses.

### Whole-genome resequencing

Whole-genome resequencing data from 31 bank voles [19] was used to obtain sequence and polymorphism information. The study sampled adult bank voles collected at Revingehed, a 43 km^2^ area 20 km east of Lund, in southern Sweden. Samples were collected at six different sites (2–8 samples per site, depending on area of each site). The maximum distance between sites was 7 km, and there are no geographical barriers (e.g. a river) between sites. Paired-end reads (Illumina Hiseq X, 2 × 150 bp) were mapped to the reference genome with BWA [51] with an average coverage of 44x and variants called with *freebayes* [52]. The raw variants were filtered in a number of steps, including for low quality, coverage and repeat overlap (see [19] for details). Haplotypes were inferred using Beagle version 4.1 [53] and coding sequences were extracted based on annotations. Sequences were edited in Geneious Prime 2020.1.1 (Biomatters) to remove gaps and errors in alignment. The curated sequence data was used to generate basic genetic diversity estimates of complement system genes using the batch mode of DnaSP 6 [54] (Additional File 1: Table S2).

### Tests for signatures of selection

We used BetaScan2 [26] to identify localised signatures of balancing selection in coding and non-coding regions. When a SNP is under balancing selection, it leads to correlations in allele frequencies between itself and neighbouring neutral SNPs, and these correlations are summarised as the β statistic, where high values indicate balancing selection. To account for differences in mutation rate across the genome, we estimated the population-scaled mutation rate using Watterson’s θ [55] in windows of 10 kb from the genotype data. For this purpose, we used bedtools 2.29.2 [56] to generate genomic intervals and PopGenome 2.7.5 [57] to calculate θ in each window. To obtain per-base pair values, the estimate in each window was divided by the window size minus the combined length of gaps and annotated repeats within the same window. BetaScan2 was run in 2 kb windows along the genome, with folded allele frequency spectrum and minimum minor allele frequency of 0.15. In each window, β values standardised by θ were calculated (β_std_) and the highest β_std_ in any window along a gene was used (β_std.max_). β values for SNPs within 1 kb of an insertion or deletion, as called by DELLY [58], were removed to avoid spurious signals. To identify outlier β_std.max_ values, the distribution of β_std.max_ in a set of control genes (n = 8465) was determined, as described in Lundberg et al., [19]. Complement genes with β_std.max_ values ≥ 95th percentile (β_std.max_ = 7.03) of the control genes’ β_std.max_ values were considered for further analyses. GNU Parallel was used to optimise the efficiency of the analysis [59].

To test for signatures of balancing selection reflected in whole coding regions we used the Hudson-Kreitman-Aguadé test (HKA; [60]) on genes that were outliers for β_std.max_. The HKA test makes use of interspecific divergence and intraspecific polymorphism data at synonymous sites to detect the presence of balancing selection. A maximum-likelihood approach to the HKA test (MLHKA; [27]) was used. The software uses polymorphism and divergence information to compare models of neutrality and selection between a set of neutrally evolving genes and the candidate gene. For the MLHKA test, a set of 20 effectively neutrally evolving genes were chosen as described in Lundberg et al., [19], with *Mus musculus* sequences as outgroup. Mouse transcripts orthologous to the neutral genes and the candidate complement genes were identified using TBLASTX. The corresponding mouse and bank vole CDS were then aligned in Geneious Prime for each gene. Alignments were checked manually for errors and indels were removed. Input parameters for each gene such as number of segregating sites (*S*) and population-scaled mutation rate (θ﻿) were calculated across all bank vole haplotypes using DnaSp. Number of divergent sites was calculated between the mouse ortholog and a randomly chosen bank vole haplotype. The program was run for each candidate gene with chain length of 100,000 for both the neutral and selection models. MLHKA uses the likelihood ratio test (LRT) to indicate significant signature of selection.

### Sliding window analysis

For genes with signatures of balancing selection as indicated by the β_std.max_ outlier analysis, we performed additional analyses across both coding and non-coding regions to identify what part of the gene was the target of selection. First, we performed sliding window analysis of nucleotide diversity and Tajima’s D using PopGenome [57] along the length of each gene, using windows of 2 kb and steps of 500 bp (except *FCNA* where windows of 500 bp and steps of 50 bp were used due to its short length). High nucleotide diversity and high positive values of Tajima’s D are expected under balancing selection [31, 32].

### Sanger sequencing

To ensure the accuracy of the whole-genome resequencing data and spot errors due to mapping and filtering, we performed Sanger sequencing of the gene with signatures of balancing selection based on the MLHKA test, *FCNA*. Primers targeting regions (~ 700 bp) around the peak of nucleotide diversity were designed to generate amplicons from a separate set of bank vole samples (n = 30). Thermocycling conditions were as follows: 94˚C for 4 min, followed by 37 cycles of denaturation at 94˚C for 30 s, annealing for 30 s at primer-specific T_m_ (Additional File 1: Table S3), extension at 72˚C for 45 s, and completed with a final extension at 72˚C for 10 min. PCR products were sequenced bidirectionally on a Genetic Analyser 3500 (Applied Biosystems) using BigDye™ terminator (Applied Biosystems). Sequences were aligned and base calls were manually checked and edited in Geneious.

### Linkage disequilibrium and haplotype networks

To investigate to what extent localised signatures of selection (identified by BetaScan and sliding window analyses of π and Tajima’s D) were in linkage disequilibrium (LD) with other parts of a gene, we constructed LD plots across the whole gene with the software LDBlockShow [61]. D**’** values were used to estimate LD and LD blocks were defined using the method by Gabriel et al., [62]. SNPs with minor allele frequency < 0.15 were filtered out.

To visualise the relationship between alleles at different SNPs in regions with signatures of balancing selection, we constructed a haplotype network from haplotypes inferred from whole-genome resequencing data and Sanger sequences. Haplotype network was constructed in popart [63] using a median joining network [64].

### Statistical analyses

Certain characteristics of genes such as their functional category (gene category) could conceivably determine if they are under balancing selection and therefore influence β_std.max_. We considered genes as “candidates of balancing selection” if they are: (a) involved in pathogen recognition (complement activation via MAMP recognition), or (b) targets of immune evasion (Additional File 1: Table S2). Targets of immune evasion were based on data in humans and primarily compiled from Lambris et al., [7], Ermert et al., [16] and from a literature survey for other complement genes that were not reported in these references. Human and rodents are infected by many related pathogens. Therefore, it is reasonable to assume that many of the targets of immune evasion identified in human pathogens are also conserved in the bank vole. To check if β_std.max_ differed between different categories, Mann-Whitney U test was used in R(v4) [65].

## Electronic supplementary material

Below is the link to the electronic supplementary material.

Table S1: List of excluded complement genes. Table S2: Summary of test statistics and categorization for all complement genes. Table S3: Primer details for Sanger sequencing.

Supplementary Figure S1: Sliding window analysis of βstd, π﻿, and Tajima’s D, haplotype network, and LD plot for the three genes that were outliers for β_std.max_

## Data Availability

Genome sequences have been deposited in SRA under BioProject PRJNA335935. The genome assembly has been deposited in GenBank with accession no. MULK00000000.1. Supplementary tables are presented in Additional File 1 and supplementary figure in Additional File 2.

## List of abbreviations

MAMPs: Microbe-associated molecular patterns
PRMs: Pattern recognition molecules
CDS: Coding sequence
MLHKA: Maximum likelihood Hudson-Kreitman-Aguadé
LD: Linkage disequilibrium
FCNA: Ficolin-A
RCA: Regulator of complement activation
MAC: Membrane attack complex
