# Supplementary material for: Balancing selection on the complement system of a wild rodent
Source: BMC Ecol Evol. 2023 May 25;23:21. doi: 10.1186/s12862-023-02122-0 (PMC10214634; doi:10.1186/s12862-023-02122-0)

Balancing selection on the complement system of a wild rodent

Mridula Nandakumar, Max Lundberg, Fredric Carlsson, Lars Råberg

Supplementary Figure S1: Sliding window analysis of β_{std}, π, and Tajima's D, haplotype network, and LD plot for the three genes that were outliers for $\beta_{std.max}$

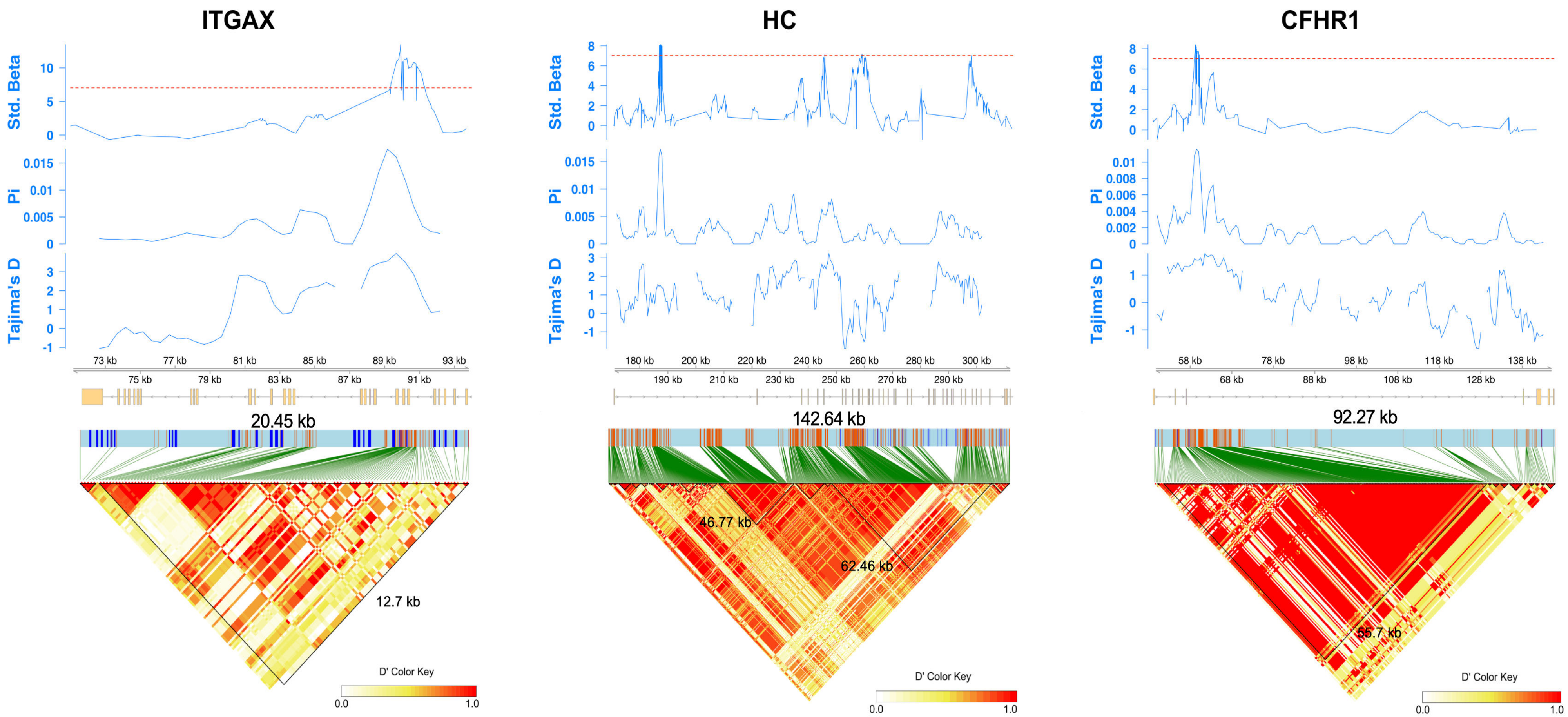

Supplement: Supplementary file 2 — Supplementary Figure S1: Sliding window analysis of βstd, π﻿, and Tajima’s D, haplotype network, and LD plot for the three genes that were outliers for βstd.max [file 12862_2023_2122_MOESM2_ESM.pdf]
